# Screening for Alcohol Use and Brief Counseling of Adults — 13 States and the District of Columbia, 2017

**DOI:** 10.15585/mmwr.mm6910a3

**Published:** 2020-03-13

**Authors:** Lela R. McKnight-Eily, Catherine A. Okoro, Khadija Turay, Cristian Acero, Dan Hungerford

**Affiliations:** ^1^Division of Birth Defects and Infant Disorders, National Center on Birth Defects and Developmental Disabilities, CDC; ^2^Division of Human Development and Disability, National Center on Birth Defects and Developmental Disabilities, CDC; ^3^Office of the Director, National Center on Emerging Zoonotic and Infectious Diseases, CDC.

Binge drinking* is a leading preventable public health problem. From 2006 to 2010, binge drinking contributed to approximately 49,000 annual deaths resulting from acute conditions (e.g., injuries and violence) (*1*). Binge drinking also increases the risk for adverse health conditions, including some chronic diseases (e.g., breast cancer) and fetal alcohol spectrum disorders (*2*). In 2004, 2013, and again in 2018, for all U.S. adults aged ≥18 years in primary care, the U.S. Preventive Services Task Force (USPSTF) recommended alcohol screening and brief intervention (alcohol SBI) or counseling for persons whose screening indicated drinking in excess of recommended limits or in ways that increase risk for poor health outcomes (*3*–*5*). However, previous CDC surveillance data indicate that patients report rarely talking to their provider about alcohol use,^†^ and alcohol SBI is traditionally delivered through conversation. CDC recently analyzed 2017 data from the Behavioral Risk Factor Surveillance System (BRFSS) survey’s five-question module, which asked adults in 13 states^§^ and the District of Columbia (DC) about the delivery of alcohol SBI during their most recent checkup in the past 2 years. Overall, 81.4% of adults (age-standardized estimate) reported being asked about alcohol use by a health professional in person or on a form during a checkup in the past 2 years, but only 37.8% reported being asked a question about binge-level alcohol consumption, which is included on USPSTF recommended instruments (*3*). Among module respondents who were asked about alcohol use at a checkup in the past 2 years and reported current binge drinking (past 30 days) at time of survey, only 41.7% were advised about the harms of drinking too much at a checkup in the past 2 years, and only 20.1% were advised to reduce or quit drinking at a checkup in the past 2 years. These findings suggest that missed opportunities remain for health care providers to intervene with patients who report binge drinking. Working to implement alcohol SBI at a systems level, including the provision of the new Healthcare Effectiveness Data Information Set (HEDIS) measure, Unhealthy Alcohol Use Screening and Follow-Up, can improve alcohol SBI’s use and benefit in primary care.

BRFSS is an ongoing state-based, random-digit–dialed telephone survey of the noninstitutionalized U.S. adult population aged ≥18 years in all 50 states, DC, and participating U.S. territories. Information is collected on various health conditions, health practices, and risk behaviors, including alcohol use. CDC analyzed 2017 data from the 13 states and DC that administered an optional alcohol SBI module. All BRFSS respondents are asked about the timing of their last routine checkup. Those who had a checkup in the past 2 years were asked alcohol SBI module questions.^¶^ All module respondents were asked four questions: 1) “You told me earlier that your last routine checkup was [within the past year/within the past 2 years]. At that checkup, were you asked in person or on a form if you drink alcohol?”; 2) “Did the health care provider ask you in person or on a form how much you drink?”; 3) “Did the health care provider specifically ask whether you drank (5 for men/4 for women) or more alcoholic drinks on an occasion?”; and 4) “Were you offered advice about what level of drinking is harmful or risky for your health?” Persons who responded affirmatively to any of the first three questions (alcohol use screening–related) were also asked “Healthcare providers may also advise patients to drink less for various reasons. At your last routine checkup, were you advised to reduce or quit your drinking?” to assess brief counseling. BRFSS assesses current binge drinking by report of drinking four (women) or five (men) or more drinks on one or more occasions during the past 30 days.

Analyses were conducted to account for BRFSS’s complex sampling design. Weighted crude and age-standardized prevalence estimates of responses to alcohol SBI module questions were calculated. Estimates were stratified by demographic characteristics and selected drinking patterns. Subanalyses were performed among alcohol SBI module respondents who indicated that they had been asked at least one of three alcohol use screening–related questions in the past 2 years and who reported current binge drinking (in the past 30 days) at time of survey. Only age-standardized estimates are included in the text of this report. Wald chi-squared tests were used to determine significant within-group differences. SUDAAN (version 11.0.3; RTI International) was used for analyses. Only significant differences are reported. The median cooperation rate** for the 14 sites was 71.7%, and median response rate was 43.8%.

Overall, 81.4% of module respondents indicated being asked by their health care provider about alcohol use in person or by form, 71.8% reported being asked how much they drink, and 37.8% reported being asked about binge drinking (Table 1). The prevalence of module respondents being asked about binge drinking was higher among males (40.1%) and persons with less than a high school diploma (46.2%) than among females (36.0%) and persons with higher levels of education (36.1% [college or technical school] to 37.1% [high school diploma]). A higher percentage of persons with a household income <200% of the federal poverty level were asked about binge drinking than were persons with an income ≥200% of the federal poverty level. Hispanic adults reported being asked about binge drinking more than other racial/ethnic groups. Prevalence of being asked about binge drinking was also higher among module respondents who reported binge drinking (47.3%) than among those who did not (36.1%).

**TABLE 1:** Weighted crude and age-standardized* percentages of U.S. adults who reported being asked an alcohol use screening–related question by a health care provider at last routine checkup in the past 2 years — Behavioral Risk Factor Surveillance System, 13 states^†^ and the District of Columbia, 2017

Characteristic	Asked about alcohol use (affirmative to question 1)			Asked how much alcohol (affirmative to question 2)			Asked about binge drinking (affirmative to question 3)		
	Sample size	Crude % (95% CI)	Age-standardized % (95% CI)	Sample size	Crude % (95% CI)	Age-standardized % (95% CI)	Sample size	Crude % (95% CI)	Age-standardized % (95% CI)
**Total**	**65,887**	**79.7 (79.0–80.5)**	**81.4 (80.7–82.1)**	**65,913**	**70.0 (69.2–70.8)**	**71.8 (70.9–72.6)**	**59,215**	**35.7 (34.8–36.6)**	**37.8 (36.9–38.8)**
**Sex**									
Male	27,725	79.7 (78.6–80.8)	81.0 (79.9–82.1)	27,663	70.1 (68.9–71.3)	71.5 (70.3–72.7)	24,942	37.9 (36.6–39.3)	40.1 (38.6–41.5)
Female	38,114	79.7 (78.7–80.7)	81.8 (80.9–82.7)	38,200	69.9 (68.8–71.0)	72.2 (71.0–73.3)	34,223	33.8 (32.5–35.0)	36.0 (34.6–37.3)
**Age group (yrs)**									
18–24	3,018	82.3 (79.7–84.7)	—	2,953	65.5 (62.3–68.5)	—	2,750	30.6 (27.7–33.6)	—
25–34	5,122	88.7 (86.9–90.3)	—	5,032	80.9 (78.7–82.9)	—	4,385	48.9 (46.2–51.7)	—
35–44	6,867	86.7 (85.1–88.2)	—	6,785	79.2 (77.1–81.1)	—	5,713	45.7 (43.2–48.3)	—
45–64	25,175	80.6 (79.4–81.8)	—	24,982	71.8 (70.4–73.1)	—	21,977	35.9 (34.4–37.3)	—
≥65	25,705	67.3 (65.7–68.9)	—	26,161	57.4 (55.8–58.9)	—	24,390	24.8 (23.4–26.3)	—
**Race/Ethnicity^§^**									
White	49,451	79.9 (79.1–80.6)	83.2 (82.5–84.0)	49,605	71.6 (70.8–72.4)	75.2 (74.3–76.1)	43,948	31.6 (30.6–32.5)	35.7 (34.5–36.9)
Black	5,993	76.2 (73.3–78.8)	77.2 (74.7–79.6)	5,967	64.9 (61.8–67.8)	65.7 (62.9–68.4)	5,632	36.5 (33.7–39.5)	36.8 (34.0–39.7)
Hispanic	5,381	83.8 (82.1–85.3)	82.4 (80.7–84.0)	5,307	70.7 (68.6–72.7)	69.7 (67.6–71.8)	4,985	46.6 (44.2–49.0)	46.9 (44.6–49.2)
A/PI	1,158	71.8 (66.4–76.5)	71.7 (66.3–76.6)	1,150	61.4 (55.9–66.5)	61.9 (56.5–67.1)	1,084	31.8 (27.0–37.1)	32.1 (27.3–37.3)
AI/AN	1,271	82.0 (77.6–85.6)	82.8 (78.6–86.3)	1,264	71.7 (66.4–76.4)	73.6 (68.9–77.9)	1,203	42.6 (36.8–48.6)	45.1 (39.8–50.5)
Other race/Multiracial	1,586	81.7 (77.7–85.2)	82.5 (78.5–85.9)	1,573	74.0 (69.1–78.4)	75.2 (70.8–79.2)	1,417	37.4 (32.0–43.1)	38.4 (33.1–43.9)
**Education level**									
Less than high school diploma	4,315	77.2 (74.9–79.3)	78.1 (75.6–80.5)	4,279	62.5 (59.7–65.1)	63.6 (60.6–66.6)	4,163	45.4 (42.5–48.3)	46.2 (43.2–49.3)
High school diploma	16,753	76.9 (75.4–78.3)	78.9 (77.4–80.2)	16,824	64.5 (62.8–66.1)	67.3 (65.6–68.9)	15,766	34.0 (32.4–35.7)	37.1 (35.3–39.0)
College or technical school	44,641	81.5 (80.6–82.4)	83.2 (82.4–84.1)	44,630	74.1 (73.1–75.1)	75.8 (74.8–76.8)	39,121	34.0 (32.9–35.1)	36.1 (34.9–37.3)
**Federal poverty level, %** ^¶^									
<100	5,896	79.0 (76.9–81.0)	78.1 (75.9–80.1)	5,850	66.6 (64.1–69.0)	65.9 (63.4–68.3)	5,570	44.0 (41.3–46.7)	44.0 (41.4–46.6)
100–199	12,773	77.5 (75.8–79.1)	80.2 (78.5–81.8)	12,842	66.6 (64.7–68.5)	69.7 (67.7–71.6)	12,049	37.8 (35.8–39.9)	41.1 (38.8–43.5)
≥200	36,949	82.3 (81.3–83.2)	83.9 (82.9–84.9)	36,873	74.5 (73.4–75.6)	75.6 (74.4–76.7)	32,219	33.7 (32.5–34.9)	36.0 (34.6–37.4)
Unknown	10,269	74.2 (72.1–76.3)	76.6 (74.3–78.7)	10,348	61.8 (59.4–64.1)	65.2 (62.5–67.8)	9,377	31.4 (29.2–33.8)	35.8 (33.0–38.8)
**Veteran**									
Yes	9,017	79.4 (77.3–81.4)	86.6 (84.2–88.7)	9,071	71.2 (68.8–73.5)	78.8 (75.0–82.1)	8,277	37.2 (35.0–39.6)	48.5 (44.4–52.7)
No	56,801	79.8 (79.0–80.5)	80.9 (80.2–81.7)	56,772	69.9 (69.0–70.7)	71.2 (70.3–72.1)	50,876	35.5 (34.5–36.5)	37.1 (36.1–38.2)
Disability status**									
Yes	20,225	75.0 (73.7–76.3)	79.4 (78.0–80.8)	20,356	64.6 (63.1–66.0)	69.4 (67.6–71.1)	19,149	34.3 (32.8–35.8)	38.8 (36.9–40.8)
No	45,143	81.6 (80.7–82.4)	82.2 (81.4–83.1)	45,055	72.2 (71.2–73.2)	72.9 (71.9–73.9)	39,614	36.4 (35.3–37.5)	37.6 (36.4–38.7)
**Health insurance coverage**									
Yes	62,282	79.8 (79.0–80.6)	81.9 (81.2–82.7)	62,369	70.5 (69.6–71.3)	72.8 (71.9–73.7)	55,905	34.9 (34.0–35.8)	37.4 (36.3–38.4)
No	3,416	78.9 (76.2–81.3)	76.9 (74.1–79.5)	3,359	65.7 (62.6–68.6)	62.8 (59.6–65.8)	3,141	45.8 (42.5–49.1)	44.5 (41.2–47.8)
**Reported current drinking**									
Yes	35,154	85.0 (84.1–85.8)	85.8 (84.9–86.6)	34,990	78.6 (77.5–79.6)	79.2 (78.2–80.2)	30,432	39.4 (38.1–40.7)	40.7 (39.3–42.1)
No	30,076	73.5 (72.3–74.6)	76.4 (75.2–77.6)	30,271	59.8 (58.5–61.1)	63.1 (61.7–64.5)	28,198	31.5 (30.3–32.8)	34.7 (33.3–36.2)
**Reported binge drinking** ^††^									
Yes	7,807	89.3 (87.9–90.6)	88.7 (87.2–90.0)	7,725	83.8 (82.1–85.4)	83.6 (81.8–85.2)	6,741	47.9 (45.2–50.5)	47.3 (44.7–50.0)
No	57,042	78.0 (77.2–78.9)	80.2 (79.4–81.0)	57,162	67.6 (66.7–68.5)	69.8 (68.8–70.8)	51,553	33.5 (32.6–34.5)	36.1 (35.0–37.2)

**Abbreviations:** AI/AN = American Indian/Alaska Native; A/PI = Asian/Pacific Islander; CI = confidence interval; FPL = federal poverty level.

* Estimates are age-standardized to the 2000 projected population for the United States.

^†^ Alabama, Alaska, Arizona, Arkansas, California, Colorado, Connecticut, Kansas, Nebraska, Nevada, New Hampshire, Tennessee, and Wisconsin.

^§^ Persons in all racial groups were non-Hispanic. Persons who self-identified as Hispanic might have been of any race.

^¶^ Poverty categories are based on the ratio of the respondent’s annual household income to the appropriate simplified 2016 federal poverty threshold (given family size: number of adults (1–14) in the household and number of children in the household) defined by the U.S. Census Bureau. This ratio is multiplied by 100 to be expressed as a percentage, and federal poverty thresholds were then used to categorize respondents into four FPL categories: 1) <100% of FPL (poor), 2) ≥100%–<200% of FPL (near poor), 3) ≥200% of FPL (not poor), and 4) unknown.

** Respondents were asked “Are you deaf or do you have serious difficulty hearing?” (hearing); “Are you blind or do you have serious difficulty seeing, even when wearing glasses?” (vision); “Because of a physical, mental, or emotional condition, do you have serious difficulty concentrating, remembering, or making decisions?” (cognition); “Do you have serious difficulty walking or climbing stairs?” (mobility); “Do you have difficulty dressing or bathing?” (self-care); and “Because of a physical, mental, or emotional condition, do you have difficulty doing errands alone such as visiting a doctor’s office or shopping?” (independent living). Respondents were identified as having one of the disability types if they answered “yes” to the relevant question. Persons who responded “yes” to at least one disability question were identified as having any disability. Persons who responded “no” to all six questions were identified as having no disability. Missing responses and respondents who answered “don’t know” or who declined to answer were excluded.

^††^ Respondents who reported consuming four or more drinks on at least one occasion during the preceding 30 days for women and five or more drinks for men. An occasion is generally defined as 2–3 hours.

Among module respondents who were asked at least one of the alcohol use screening–related questions at a checkup in the past 2 years and reported current binge drinking (past 30 days) at time of survey, only 41.7% were advised about the harms of drinking too much at a checkup in the past 2 years, and only 20.1% were advised to reduce or quit drinking at a checkup in the past 2 years (Table 2). Among module respondents who were asked at least one of the alcohol use screening–related questions and reported current binge drinking in the past 30 days at time of survey, the prevalence of being advised to reduce drinking at a checkup in the past 2 years was higher among males (24.1%), persons with a disability (28.2%), persons with less than a high school education (38.2%), persons with income <100% of the federal poverty level (37.5%), and those without health insurance coverage (36.2%) than among their counterparts. Prevalence was also higher among Hispanic adults (28.5%) than among white adults (16.8%).

**TABLE 2:** Weighted crude and age-standardized* estimates of being advised about harmful or risky drinking levels and to reduce the level of drinking, among U.S. adults who reported being asked an alcohol use screening–related question by a health care provider at last routine checkup in the past 2 years and reported current binge drinking in the past 30 days at time of survey^†^ — Behavioral Risk Factor Surveillance System, 13 states^§^ and the District of Columbia, 2017

Characteristic	Adults who were asked an alcohol use screening–related question^¶^ and reported current binge drinking in the past 30 days at time of survey					
	Advised about level of drinking harmful/risky to health^¶^			Advised to reduce drinking^¶^		
	Sample size	Crude % (95% CI)	Age–standardized % (95% CI)	Sample size	Crude % (95% CI)	Age–standardized % (95% CI)
**Total**	**6,811**	**41.8 (39.1–44.4)**	**41.7 (39.0–44.4)**	**6,943**	**20.6 (18.6–22.6)**	**20.1 (18.2–22.2)**
**Sex**						
Male	3,924	47.4 (44.0–50.8)	46.7 (43.2–50.3)	4,004	24.9 (22.3–27.8)	24.1 (21.5–26.8)
Female	2,883	32.7 (28.9–36.7)	33.8 (29.9–37.9)	2,935	13.6 (11.0–16.5)	13.7 (10.9–17.1)
**Age group (yrs)**						
18–24	655	43.2 (35.6–51.2)	—	665	16.6 (12.2–22.2)	—
25–34	1,187	42.4 (37.1–47.8)	—	1,214	20.8 (16.8–25.4)	—
35–44	1,186	37.6 (31.5–44.0)	—	1,213	17.1 (13.5–21.4)	—
45–64	2,754	42.7 (38.8–46.7)	—	2,812	25.9 (22.4–29.8)	—
≥65	1,029	43.3 (35.2–51.7)	—	1,039	15.7 (10.9–22.3)	—
Race/Ethnicity**						
White	5,046	40.9 (38.1–43.8)	40.9 (38.0–43.9)	5,161	16.8 (15.0–18.9)	16.8 (14.9–19.0)
Black	511	43.0 (34.2–52.3)	44.8 (35.7–54.4)	514	20.1 (14.5–27.1)	19.9 (15.0–25.9)
Hispanic	720	42.6 (36.3–49.1)	42.6 (35.2–50.4)	729	28.8 (23.4–34.8)	28.5 (23.4–34.2)
A/PI	105	45.9 (29.3–63.4)	50.5 (33.9–67.1)	104	19.9 (10.7–33.9)^††^	N/A**^§§^**
AI/AN	161	58.7 (41.6–74.0)	57.8 (44.8–69.7)	163	N/A^§§^	27.9 (17.2–41.9)^††^
Other race/Multiracial	189	35.6 (24.2–49.0)	34.9 (24.4–47.1)	190	23.6 (13.8–37.4)^††^	24.6 (15.1–37.5)^††^
**Education level**						
Less than high school diploma	340	61.9 (52.5–70.4)	63.8 (54.4–72.3)	343	42.4 (33.2–52.2)	38.2 (30.0–47.1)
High school diploma	1,659	40.4 (35.1–45.8)	40.5 (35.5–45.8)	1,682	22.0 (18.1–26.4)	21.1 (17.6–25.2)
College or technical school	4,806	38.5 (35.7–41.4)	38.4 (35.5–41.4)	4,912	16.0 (14.0–18.2)	16.0 (13.9–18.3)
**Federal poverty level, %** ^¶¶^						
<100	578	44.9 (37.3–52.8)	41.9 (34.9–49.3)	583	36.2 (28.9–44.1)	37.5 (30.8–44.8)
100–199	1,076	47.9 (41.0–54.9)	47.9 (41.1–54.7)	1,088	21.8 (17.2–27.2)	21.5 (17.6–26.1)
≥200	4,532	39.0 (36.0–42.2)	39.4 (36.3–42.7)	4,632	16.5 (14.5–18.8)	16.3 (14.2–18.8)
Unknown	625	42.4 (33.6–51.7)	39.8 (33.3–46.8)	640	21.7 (15.5–29.4)	23.6 (17.6–30.9)
**Veteran**						
Yes	856	52.1 (45.1–59.1)	53.5 (45.8–61.1)	873	21.3 (16.3–27.4)	21.5 (16.1–28.0)
No	5,951	40.6 (37.8–43.4)	40.8 (37.8–43.9)	6,066	20.5 (18.4–22.7)	19.8 (17.7–22.1)
Disability status***						
Yes	1,451	50.1 (44.3–56.0)	49.2 (43.7–54.7)	1,472	28.7 (24.1–33.9)	28.2 (23.6–33.3)
No	5,330	39.5 (36.6–42.5)	39.3 (36.3–42.3)	5,440	18.4 (16.3–20.7)	17.8 (15.8–19.9)
**Health insurance coverage**						
Yes	6,287	41.6 (38.8–44.4)	41.4 (38.6–44.3)	6,407	19.3 (17.3–21.4)	18.9 (16.9–21.0)
No	507	44.3 (36.9–52.1)	48.6 (40.7–56.6)	518	33.1 (26.1–41.0)	36.2 (28.2–45.1)

**Abbreviations:** AI/AN = American Indian/Alaska Native; A/PI = Asian/Pacific Islander; CI = confidence interval; FPL = federal poverty level; N/A = not available.

* Estimates are age-standardized to the 2000 projected population for the United States.

**^†^** Respondents who reported consuming four or more drinks on at least one occasion during the preceding 30 days for women and five or more drinks for men. An occasion is generally defined as 2–3 hours.

^§^ Alabama, Alaska, Arizona, Arkansas, California, Colorado, Connecticut, Kansas, Nebraska, Nevada, New Hampshire, Tennessee, and Wisconsin.

^¶^ At a checkup in the past 2 years.

** Persons in all racial groups were non-Hispanic; persons who self-identified as Hispanic might have been of any race.

^††^ Relative standard error = 0.20–0.30.

**^§§^** Estimate not available because relative standard error >0.30.

^¶¶^ Poverty categories are based on the ratio of the respondent’s annual household income to the appropriate simplified 2016 federal poverty threshold (given family size: number of adults (1–14) in the household and number of children (≥0) in the household) defined by the U.S. Census Bureau. This ratio is multiplied by 100 to be expressed as a percentage, and federal poverty thresholds were then used to categorize respondents into four FPL categories: 1) <100% of FPL (poor), 2) ≥100% to <200% of FPL (near poor), 3) ≥200% of FPL (not poor), and 4) unknown.

*** Respondents were asked “Are you deaf or do you have serious difficulty hearing?” (hearing); “Are you blind or do you have serious difficulty seeing, even when wearing glasses?” (vision); “Because of a physical, mental, or emotional condition, do you have serious difficulty concentrating, remembering, or making decisions?” (cognition); “Do you have serious difficulty walking or climbing stairs?” (mobility); “Do you have difficulty dressing or bathing?” (self-care); and “Because of a physical, mental, or emotional condition, do you have difficulty doing errands alone such as visiting a doctor’s office or shopping?” (independent living). Respondents were identified as having one of the disability types if they answered “yes” to the relevant question. Persons who responded “yes” to at least one disability question were identified as having any disability. Persons who responded “no” to all six questions were identified as having no disability. Missing responses and respondents who answered “don’t know” or who declined to answer were excluded.

## Discussion

In 2017, although 81% of U.S. adults reported being asked by their health care provider about alcohol use, only 38% reported being asked about binge drinking during a checkup in the past 2 years, based on BRFSS data from 13 states and DC. Fewer than half (42%) of module respondents who were asked about alcohol use at a checkup in the past 2 years and reported current binge drinking (past 30 days) at time of survey were advised of harmful drinking levels; almost 80% (four of five persons) received no advice to reduce their drinking (only 20% were advised to reduce their drinking). Previous overall 2014 estimates, using the BRFSS alcohol SBI module from 17 states and DC (*6*), were similar to overall 2017 findings, but not directly comparable because of differences in states repeating the module in 2014 and 2017 (only five states implemented the module both years). State-level trend analysis might occur in future reports. An assessment of binge-level consumption is included on USPSTF-recommended screening tools (*5*).

Screening alone is not effective at reducing binge drinking (*5*). Brief counseling involves feedback based on screening results, a conversation about the dangers of excessive drinking on the patient’s health, and development of a plan to reduce drinking if the patient chooses to do so (*5*,*7*). Behavioral counseling is a necessary component of alcohol SBI for reduction in consumption and adherence to drinking limits (*5*). Persons with dependence are to be referred to treatment, but referral might not occur, or patients might not accept the referral, obtain treatment, or respond to treatment (*5*,*7*). The Substance Abuse and Mental Health Services Administration, which has long promoted SBI through grant programs, has a treatment locator^††^ to assist with the referral process. A 2018 USPSTF review found that “Among adults identified through screening, counseling interventions to reduce unhealthy alcohol use were associated with reductions in alcohol use (by a mean of 1.6 drinks/wk) and in the odds of exceeding recommended drinking limits (by 40%) and heavy use episodes (by 33%) at 6 to 12 months of follow-up….Among pregnant women, counseling interventions were associated with an odds ratio of 2.26 for remaining abstinent from alcohol during pregnancy.” (*5*).

The demographic differences in this report might be a consequence of some adults having more contact with health systems, such as those with a disability (*8*) or veterans, which could increase their likelihood of receipt of alcohol SBI. In addition, screening practices might vary in health care systems that have systematically implemented alcohol SBI (e.g., U.S. Department of Veterans Affairs and federally qualified health centers) (*9*).

Health system changes, such as the acceptance of a new 2018 HEDIS measure: Unhealthy Alcohol Use Screening and Follow-Up,^§§^ might increase the provision of alcohol SBI. Further, federal agencies have promoted broad implementation of alcohol SBI, including CDC’s funding initiatives to organizations working on fetal alcohol spectrum disorder,^¶¶^ the development of training and implementation resources,*** and cross-agency, medical, and private sector partnerships.

The 2015–2020 Dietary Guidelines for Americans recommends that if alcohol is consumed, it should be in moderation (up to one drink a day for women, two for men) and only by adults of legal drinking age.^†††^ The 2015–2020 Dietary Guidelines for Americans and the National Institute for Alcohol Abuse and Alcoholism also indicate or advise that some persons should not drink alcohol at all, including pregnant women (*10*) or those who might be pregnant or persons who have certain medical conditions or are taking medications that can interact with alcohol (*10*).

The findings in this report are subject to at least four limitations. First, BRFSS data are self-reported, which can result in recall and social desirability biases around the period of recall for the checkup. Second, data in this report were from 14 sites, and thus, these results cannot be used to estimate the prevalence of alcohol SBI across all states and territories. Third, although respondents indicated current binge drinking in response to a BRFSS survey question, whether they reported binge drinking to their health care provider at time of checkup in the past 2 years is unknown; many respondents reported not being asked about binge drinking at a checkup in the past 2 years. Finally, the survey median response rate was only 43.8%, which increases the possibility of response bias.

Binge drinking among U.S. adults continues to be a leading preventable cause of considerable morbidity and mortality (*1*). Alcohol SBI is an effective clinical preventive service for reducing excess alcohol use,^§§§^ including binge consumption (*3*,*5*,*7*). This report suggests that alcohol SBI is not being fully implemented as recommended. If alcohol SBI is implemented as recommended by USPSTF (*3*,*5*,*7*) and coupled with population-level interventions recommended by the U.S. Community Preventive Services Task Force for the prevention of excessive drinking (e.g., increasing alcohol taxes and regulating alcohol outlet density^¶¶¶^), an opportunity exists to also reduce alcohol-related morbidity and mortality. Working to implement alcohol SBI at a systems level, including the provision of the new HEDIS measure, Unhealthy Alcohol Use Screening and Follow-Up, can improve alcohol SBI’s use and benefit in primary care.

**Summary**

**What is already known about this topic?**

Binge drinking increases the risk for adverse health conditions and death. Alcohol screening and brief intervention (SBI), recommended by the U.S. Preventive Services Task Force (USPSTF) for all adults in primary care, is effective in reducing binge drinking.

**What is added by this report?**

In 2017, 81% of survey respondents were asked by their health care provider about alcohol consumption and 38% about binge drinking at a checkup in the past 2 years. Among those asked about alcohol use and who reported current binge drinking, 80% received no advice to reduce their drinking.

**What are the implications for public health practice?**

Implementation of alcohol SBI as recommended by USPSTF, coupled with population-level evidence-based interventions, can reduce binge drinking among U.S. adults.

## Footnotes

* The National Institute on Alcohol Abuse and Alcoholism defines binge drinking as a pattern of drinking that brings blood alcohol concentration levels to 0.08 g/dL. This typically occurs after four drinks for women and five drinks for men, in about 2 hours. https://pubs.niaaa.nih.gov/publications/Newsletter/winter2004/Newsletter_Number3.pdf.

† https://www.cdc.gov/vitalsigns/alcohol-screening-counseling/index.html.

§ Alabama, Alaska, Arizona, Arkansas, California, Colorado, Connecticut, Kansas, Nebraska, Nevada, New Hampshire, Tennessee, and Wisconsin.

¶ The module lead-in question was “Healthcare providers may ask during routine checkups about behaviors like alcohol use, whether you drink or not. We want to know about their questions.”

** The cooperation rate is the number of complete and partial complete interviews divided by the number of contacted and eligible respondents.

†† https://findtreatment.samhsa.gov/.

§§ The 2018 Healthcare Effectiveness Data Information Set (HEDIS) measure, Unhealthy Alcohol Use Screening and Follow-Up, was approved as a first-year measure in June 2017 by the National Committee for Quality Assurance as a 2018 HEDIS measure. The testing and submission of this measure into HEDIS was supported by the Substance Abuse and Mental Health Services Administration. The measure uses standardized tools for alcohol SBI and for those who screen positive, the percentage who receive brief counseling or other follow-up within 2 months of the positive screen is documented. https://www.ncqa.org/hedis/reports-and-research/hedis-measure-unhealthy-alcohol-use-screening-and-follow-up/.

¶¶ https://www.cdc.gov/ncbddd/fasd/alcohol-screening.html.

*** https://www.cdc.gov/ncbddd/fasd/documents/alcoholsbiimplementationguide.pdf.

††† https://health.gov/dietaryguidelines/2015/.

§§§ https://www.cdc.gov/alcohol/fact-sheets/binge-drinking.htm.

¶¶¶ https://www.thecommunityguide.org/topic/excessive-alcohol-consumption.
